# Effects of SGLT2 inhibitors on the onset of esophageal varices and extrahepatic cancer in type 2 diabetic patients with suspected MASLD: a nationwide database study in Japan

**DOI:** 10.1007/s00535-024-02158-z

**Published:** 2024-10-11

**Authors:** Takumi Kawaguchi, Yoshiyuki Fujishima, Daisuke Wakasugi, Fusayo Io, Yuri Sato, Saeko Uchida, Yukiko Kitajima

**Affiliations:** 1 Division of Gastroenterology, Department of Medicine, Kurume University School of Medicine, 67 Asahi-machi, Kurume, 830-0011 Japan; 2 Taisho Pharmaceutical Co., Ltd., Tokyo, Japan

**Keywords:** Metabolic dysfunction-associated steatotic liver disease, Sodium–glucose co-transporter 2 inhibitors, Esophageal varices, Extrahepatic malignancy, Real-world evidence

## Abstract

**Background & aim:**

SGLT2 inhibitors (SGLT2i) improve hepatic steatosis in patients with type 2 diabetes mellitus (T2DM) and MASLD. We aimed to investigate the impact of SGLT2i on the incidence of liver-related events and extrahepatic cancer compared to DPP4 inhibitors (DPP4i) in patients with T2DM and suspected MASLD using a medical claims database in Japan.

**Methods:**

We conducted a retrospective study using a Japanese medical claims database. Among patients with T2DM who were prescribed SGLT2i or DPP4i (*n* = 1,628,656), patients with suspected MASLD were classified into SGLT2i (*n* = 4204) and DPP4i (*n* = 4204) groups. Effects of SGLT2i on the following outcomes were compared to DPP4i: (1) changes in HbA1c and ALT levels after 6 months, (2) changes in hepatic fibrosis index, and (3) the incidence of liver-related events/extrahepatic cancer over 12 months.

**Results:**

After 6 months, DPP4i significantly decreased HbA1c levels compared to SGLT2i. In contrast, SGLT2i significantly decreased ALT levels compared to DPP4i. SGLT2i significantly decreased FIB-4 index compared to DPP4i over 12 months. Although no significant difference was observed in the incidence of overall liver-related events between the two groups, SGLT2i significantly reduced the incidence of esophageal varices (HR 0.12, 95%CI 0.01–0.95, *P* = 0.044). Moreover, SGLT2i significantly suppressed the incidence of extrahepatic cancer (HR 0.50, 95%CI 0.30–0.84, *P* = 0.009) compared to DPP4i.

**Conclusion:**

SGLT2i was more beneficial than DPP4i in improving the hepatic inflammation and fibrosis indices. Moreover, SGLT2i suppressed the incidence of esophageal varices and extrahepatic cancer compared to DPP4i. SGLT2i may suppress life-threatening events in patients with T2DM and suspected MASLD.

**Supplementary Information:**

The online version contains supplementary material available at 10.1007/s00535-024-02158-z.

## Introduction

Hepatic steatosis is highly prevalent in patients with type 2 diabetes mellitus (T2DM) [1]. The American Diabetes Association recently updated clinical practice recommendations and proposed screening for non-alcoholic fatty liver disease (NAFLD) in patients with T2DM [2]. Similarly, in patients with NAFLD, the co-occurrence of metabolic dysfunction is a well-known risk factor for disease progression [3–5]. Recently, metabolic dysfunction-associated steatotic liver disease (MASLD) was chosen to replace NAFLD with a multi-society Delphi consensus statement led by three large pan-national liver associations [6]. In particular, T2DM is a potent prognostic risk factor[3–5]; therefore, anti-diabetic medication is an important therapeutic strategy in patients with MASLD.

Sodium–glucose co-transporter 2 inhibitors (SGLT2i) are a widely used anti-diabetic medication. A meta-analysis demonstrated that SGLT2i improves not only glucose and lipid metabolisms but also serum hepatic enzyme levels including alanine aminotransferase (ALT) in patients with MASLD [7]. Meta-analysis also demonstrated that SGLT2i improves hepatic steatosis and fibrosis in patients with MASLD [8]. These hepato-protective effects of SGLT2i have been reported to be superior to those of dipeptidyl peptidase 4 inhibitors (DPP4i) [9, 10]. However, it remains unclear whether SGLT2i exerts inhibitory effects on the incidence of liver-related events, including esophageal varices, in patients with MASLD accompanied by T2DM.

Besides liver-related events, extrahepatic cancer is a leading cause of mortality in patients with MASLD worldwide [11, 12]. Unlike liver-related events, extrahepatic cancer develops regardless of the degree of hepatic fibrosis [12]. It is difficult to screen for various extrahepatic cancers in all patients with MASLD, and prevention is critical for the management of patients with MASLD. Basic studies have demonstrated that SGLT2i exerts direct anti-tumor effects by inhibiting glucose uptake and ATP production in various cancer cells [13–15]. However, clinical studies have reported conflicting results regarding the anti-tumor effects of SGLT2i[16], and a consensus has not yet been reached. Thus, a large-scale real-world database study is required to explore the preventive effects of SGLT2i on extrahepatic cancer in patients with MASLD.

The universal health insurance system covers the medical care for all citizens and provides real-world data. The healthcare system has been introduced in several countries including England, Sweden, and Japan. Medical Data Vision (MDV) is a large Japanese administrative claims database of approximately 42.32 million patients in 474 hospitals participating in the Diagnosis Procedure Combination (DPC) system [17]. MDV can monitor the same patients’ information throughout outpatient and inpatient care over time. The database includes information about diagnosed diseases, biochemical examinations, and prescribed medications. MDV has provided real-world evidence for comorbidities in patients with MASLD in Japan [17]. MDV has also demonstrated the superiority of SGLT2i over DPP4i in reducing cardiovascular disease events [18]. Furthermore, MDV has allowed us to investigate the incidence and treatment of various cancers [19, 20].

This study aimed to investigate the effects of SGLT2i on hepatic inflammation and fibrosis by comparing DPP4i in patients with T2DM and suspected MASLD using a nationwide medical claims database. We also investigated the effects of SGLT2i on the incidence of liver-related events, including esophageal varices, by comparing DPP4i. We further investigated the effects of SGLT2i on the incidence of extrahepatic cancer in patients with T2DM and suspected MASLD.

## Methods

### Study design, data source, and ethics

This was a retrospective cohort study using data extracted from the MDV, covering the period from October 1, 2013 to October 31, 2022. The MDV provides electronic medical insurance claims data from the DPC hospitals in Japan. MDV contains information on about 42.32 million patients as of the end of December 2022. Data collected included patient information, including age, sex, diagnosed diseases, biochemical examinations, and prescribed drugs.

This study was reviewed by the Research Institute of Healthcare Data Science, a general incorporated association (RI2022013). In addition, the study was conducted in accordance with the Declaration of Helsinki, ethical guidelines for medical research involving human subjects, and the Personal Information Protection Act.

### Subjects

**Fig. 1:**
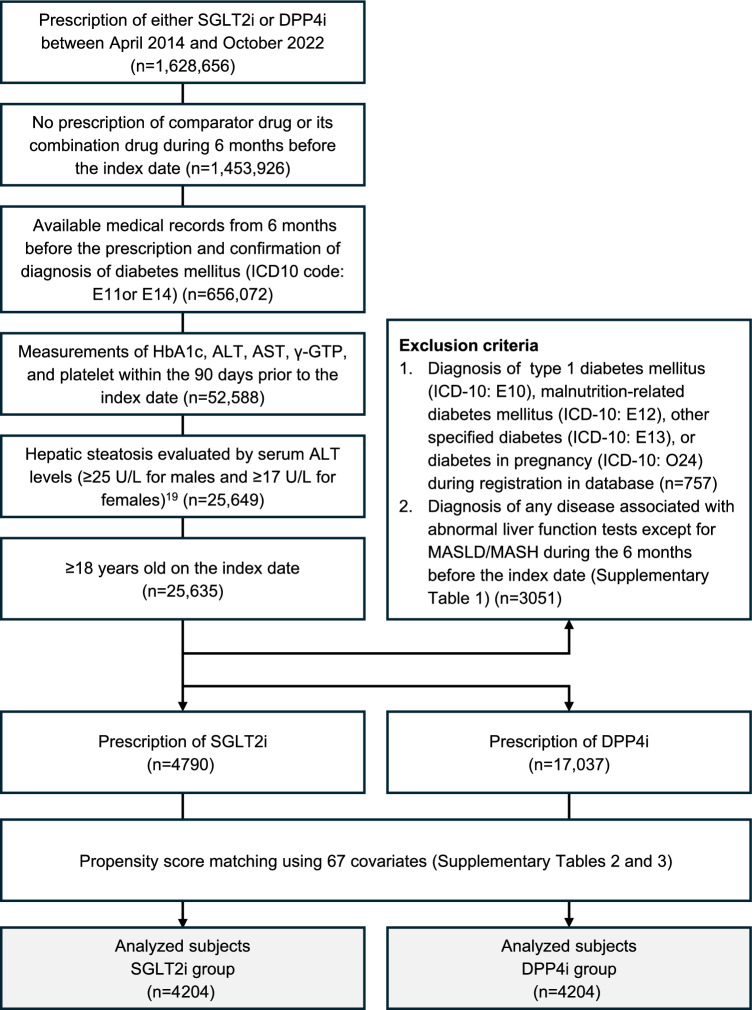
Flowchart for the selection of the study population

We enrolled patients with T2DM who were prescribed either SGLT2i or DPP4i between April 1, 2014 and October 31, 2022 (*n* = 1,628,656) (Fig. 1). Enrollment was defined based on an index date, which is the initial prescription date for either SGLT2i or DPP4i.

We selected patients according to the following inclusion and exclusion criteria: (i) patients who were prescribed either SGLT2i or DPP4i; (ii) no prescription of comparator drug (DPP4i for SGLT2i prescriptions or SGLT2i for DPP4i prescriptions) or its combination drug during 6 months before the index day; (iii) medical records were available from 6 months before the prescription and a confirmed diagnosis of T2DM (International Classification of Diseases, 10th Revision [ICD-10]: E11) or unspecified diabetes mellitus (ICD-10: E14); (iv) the following measurements were taken within 90 days before the index date: hemoglobin A1c (HbA1c), ALT, aspartate aminotransferase (AST), gamma-glutamyl transpeptidase (γ-GTP), and platelets; (v) baseline serum ALT levels of ≥ 25 U/L for males and ≥ 17 U/L for females; (vi) ≥ 18 years of age on the index day. The MDV database does not include information on body mass index, abdominal circumference, and imaging including abdominal ultrasound. Therefore, we defined suspected MASLD by the serum ALT levels (≥ 25 U/L for males and ≥ 17 U/L for females), as previously described [21].

Exclusion criteria were (i) diagnosis of type 1 diabetes mellitus (ICD-10: E10), malnutrition-related diabetes mellitus (ICD-10: E12), other specified diabetes (ICD-10: E13), or diabetes in pregnancy (ICD-10: O24) during the database enrollment period; and (ii) a diagnosis of any disease associated with abnormal liver function tests except for MASLD/MASH during 6 months before the index date (Supplementary Table 1).

Eligible patients were classified into SGLT2i or DPP4i groups according to the medication on the index date. Both groups underwent a 1:1 propensity score matching, forming a population analysis using 67 covariates (Supplementary Tables 2 and 3).

Termination of the prescription date is defined as follows: i) the end of the observation period, ii) the development of the event, iii) prescription of another comparison drug (SGLT2i or DPP4i) including combination drugs, and iv) no prescription for more than 31 days since the date obtained by adding the number of days of prescription to the date of last prescription of SGLT2i or DPP4i.

### Outcomes

The outcomes are (1) changes in HbA1c and ALT levels 6 months after the prescription of either SGLT2i or DPP4i, (2) changes in hepatic fibrosis indexes including platelet, FIB-4 index, Forns index, AST/ALT ratio, and Diabetes Mellitus-hepatocellular carcinoma (DM-HCC) risk score from baseline over 12 months, (3) the incidence of newly diagnosed liver-related events (liver cancer (ICD-10: C22), cirrhosis (ICD-10: K74.6, K76.7, R18), liver failure (ICD-10: K72), esophageal varices (ICD-10: I85, I86.4)), extrahepatic cancer (ICD-10: C00-C21, C23-C96), and cardiovascular events (myocardial infarction (ICD-10: I21), angina (ICD-10: I20), atrial fibrillation (ICD-10: I48), stroke (ICD-10: I60-I64), heart failure (ICD-10: I50), cardiac arrest (ICD-10: I46)) during 12 months (Supplementary Table 1). The formula for calculating the hepatic fibrosis indices and the DM-HCC risk score is described in Supplementary Table 4.

### Statistical analysis

Statistical analysis of this study was performed independently by Datack Inc. (Tokyo, Japan). Researchers and Taisho Pharmaceutical personnel were not included in the statistical analysis.

Our primary objective is to investigate the risk for the incidence of liver-related events and extrahepatic cancer in comparable SGLT2i and DPP4i groups. Many factors interact in a complex manner in the development of life-threatening events. In accordance with previous studies examining the efficacy of SGLT2i [22–24], we chose propensity score matching rather than the stepwise method because of the following 3 advantages: bias control, model flexibility, and stability of results [25, 26].

Based on the recent theoretical principles of confounder selection [27], propensity score matching was performed with 67 covariates including sex, age, year of index date, presence or absence of diabetes medication prescription (by drug class) at the index date, concomitant diseases, such as hypertension, dyslipidemia, cirrhosis, and the presence of disease, as defined by the Charlson Comorbidity Index, baseline laboratory test values, such as HbA1c, ALT, AST, γ-GTP, platelets, and estimated glomerular filtration rate (eGFR) (Supplementary Tables 2 and 3).

Logistic regression analysis was performed using the nearest neighbor method, with CALIPER set at 0.2 times a standard deviation. Balance was assessed using the standardized mean difference (SMD). For laboratory values, we used the Multiple Imputation by Chained Equations (MICE) method to impute missing data for eGFR, total cholesterol, and triglyceride. Then, we calculated the Forns index and DM-HCC risk score.

For the between-groups comparison, group variances and corresponding 95% confidence intervals (CI) were calculated based on the change from baseline to 6 months for patients with available laboratory values in the analyzed population, using Welch's test with a significance level set at *p* < 0.05. For the time-course comparison over 12 months, a linear mixed model was used to estimate the change in each laboratory test value over time for each time point, and intra-/intergroup differences and 95%CI were calculated using the R package (lmerTest). Hazard ratios with 95%CI were calculated using Cox regression analysis to compare the risks of liver-related events, extrahepatic cancer, and cardiovascular events between the SGLT2i and DPP4i groups. Probability of esophageal varices and extrahepatic cancer was calculated using the Kaplan–Meier method and analyzed using the log-rank test. All data analyses were conducted using the Amazon Athena engine version 3 (Amazon.com, Inc.), and statistical analysis was performed using R (version 4.2.1 (The R Project for Statistical Computing).

## Results

### Patients enrollment

The patient selection process is summarized in Fig. 1. From the patients prescribed either SGLT2i or DPP4i during the registration period (*n* = 1,628, 656), we selected the subjects for analysis according to the inclusion and exclusion criteria. Eligible patients were divided into SGLT2i (*n* = 4790) or DPP4i (*n* = 17,037) groups. After propensity score matching at a 1:1 ratio, 4204 patients in each group were included in the analysis.

### Patients’ characteristics

Before propensity score matching, there were significant differences in the patient characteristics between the SGLT2i and DPP4i groups (Table 1). The SGLT2i group was younger and had a higher prevalence of concomitant GLP-1 receptor agonists use and dyslipidemia than the DPP4i group. Furthermore, the SGLT2i group showed higher triglyceride levels and lower Forns index and DM-HCC risk score (Table 1).

**Table 1:** Patients’ characteristics of the original cohort and propensity score-matched cohort

Variables	Original cohort			Propensity score-matched cohort		
	SGLT2i, *n* = 4,790	DPP4i, *n* = 17,037	SMD	SGLT2i, *n* = 4,204	DPP4i, *n* = 4,204	SMD
Age, Mean (SD)	61 (14)	70 (13)	– 0.62	62 (14)	62 (14)	0.01
Sex (Female)	1837 (38%)	8002 (47%)	– 0.17	1638 (39%)	1606 (38%)	0.02
Concomitant diabetes medications						
Insulins	1056 (22%)	4116 (24%)	– 0.05	830 (20%)	827 (20%)	0
Sulfonylureas	497 (10%)	2664 (16%)	– 0.16	405 (9.6%)	414 (9.8%)	– 0.01
Biguanides	1612 (34%)	4344 (25%)	0.18	1313 (31%)	1327 (32%)	– 0.01
Glitazones	185 (3.9%)	668 (3.9%)	0	148 (3.5%)	143 (3.4%)	0.01
α-glucosidase inhibitors	378 (7.9%)	1926 (11%)	– 0.12	310 (7.4%)	330 (7.8%)	– 0.02
Glinides	176 (3.7%)	820 (4.8%)	– 0.06	143 (3.4%)	142 (3.4%)	0
GLP-1 receptor agonists	707 (15%)	184 (1.1%)	0.52	252 (6.0%)	175 (4.2%)	0.08
Other antidiabetics	2 (< 0.1%)	3 (< 0.1%)	0.01	1 (< 0.1%)	1 (< 0.1%)	0
Concomitant medications other than diabetes medications						
Anti-hypertensives	304 (6.3%)	784 (4.6%)	0.08	261 (6.2%)	244 (5.8%)	0.02
Diuretics	1135 (24%)	3351 (20%)	0.1	972 (23%)	916 (22%)	0.03
Beta-blockers	1263 (26%)	3212 (19%)	0.18	1096 (26%)	1046 (25%)	0.03
Calcium antagonists	1979 (41%)	7962 (47%)	– 0.11	1763 (42%)	1769 (42%)	0
Angiotensin-converting enzyme inhibitors/ angiotensin receptor blockers	2396 (50%)	7438 (44%)	0.13	2055 (49%)	2028 (48%)	0.01
Statins	2299 (48%)	7505 (44%)	0.08	1981 (47%)	1,950 (46%)	0.01
Vitamin E	14 (0.3%)	23 (0.1%)	0.03	13 (0.3%)	13 (0.3%)	0
Fibrates	268 (5.6%)	628 (3.7%)	0.09	219 (5.2%)	219 (5.2%)	0
Other anti-hyperlipidemics	663 (14%)	1543 (9.1%)	0.15	566 (13%)	570 (14%)	0
Concomitant diseases						
Hypertension	3173 (66%)	10497 (62%)	0.1	2758 (66%)	2742 (65%)	0.01
Dyslipidemia	2982 (62%)	8401 (49%)	0.26	2569 (61%)	2583 (61%)	– 0.01
Chronic hepatitis	99 (2.1%)	213 (1.3%)	0.06	77 (1.8%)	86 (2.0%)	– 0.02
Liver cirrhosis	40 (0.8%)	151 (0.9%)	– 0.01	35 (0.8%)	37 (0.9%)	– 0.01
Biochemical examination						
HbA1c (%)	7.98 (1.76)	7.79 (1.64)	0.11	7.92 (1.74)	7.98 (1.74)	– 0.03
AST (U/L)	43 (128)	40 (131)	0.03	44 (137)	44 (245)	0
ALT (U/L)	51 (58)	44 (67)	0.11	51 (59)	50 (104)	0
Platelet (×10^3^/µL)	23 (7)	22 (8)	0.09	23 (7)	22 (7)	0.03
γ-GTP (U/L)	73 (91)	72 (118)	0.01	74 (94)	74 (98)	0
Triglyceride (mg/dL)	193 (154)	165 (122)	0.2	190 (153)	190 (160)	0
Total cholesterol (mg/dL)	192 (39)	188 (38)	0.12	192 (39)	192 (40)	0.01
eGFR (mL/min/1.73m^2^)	72 (25)	69 (27)	0.1	72 (24)	72 (26)	– 0.02
Hepatic fibrosis indexes and HCC risk score						
FIB-4 index	1.91 (2.69)	2.25 (3.36)	– 0.11	1.97 (2.77)	1.90 (2.34)	0.03
Forns index	5.49 (1.83)	6.09 (1.75)	– 0.33	5.56 (1.83)	5.59 (1.81)	– 0.01
AST/ALT ratio	0.89 (0.50)	0.96 (0.49)	– 0.13	0.91 (0.52)	0.89 (0.47)	0.03
DM-HCC risk score	14 (8)	17 (8)	– 0.31	14 (9)	14 (8)	0.02

Data are expressed by mean ± SD, number, percentage, or SMD

*SD* standard deviation, *SMD* standardized mean difference, *GLP-1* glucagon-like peptide-1, *HbA1c* hemoglobin A1c, *AST* aspartate aminotransferase, *ALT* alanine aminotransferase, *γ-GTP* γ-glutamyl transferase, *eGFR* estimated glomerular filtration rate, *FIB-4 index* Fibrosis 4 index, *DM-HCC risk score* diabetes mellitus hepatocellular carcinoma risk score

After propensity score matching, all variables included in the propensity score analysis exhibited an absolute SMD of less than 0.10. Both groups had a mean age of 62 years and a similar sex distribution. There was no significant difference in GLP-1 receptor agonists use between the SGLT2i and DPP4i groups. No significant differences were observed in HbA1c levels, ALT levels, or all hepatic fibrosis indices between the two groups (Table 1).

### Effects of SGLT2i on HbA1c and ALT levels compared to DPP4i

**Fig. 2:**
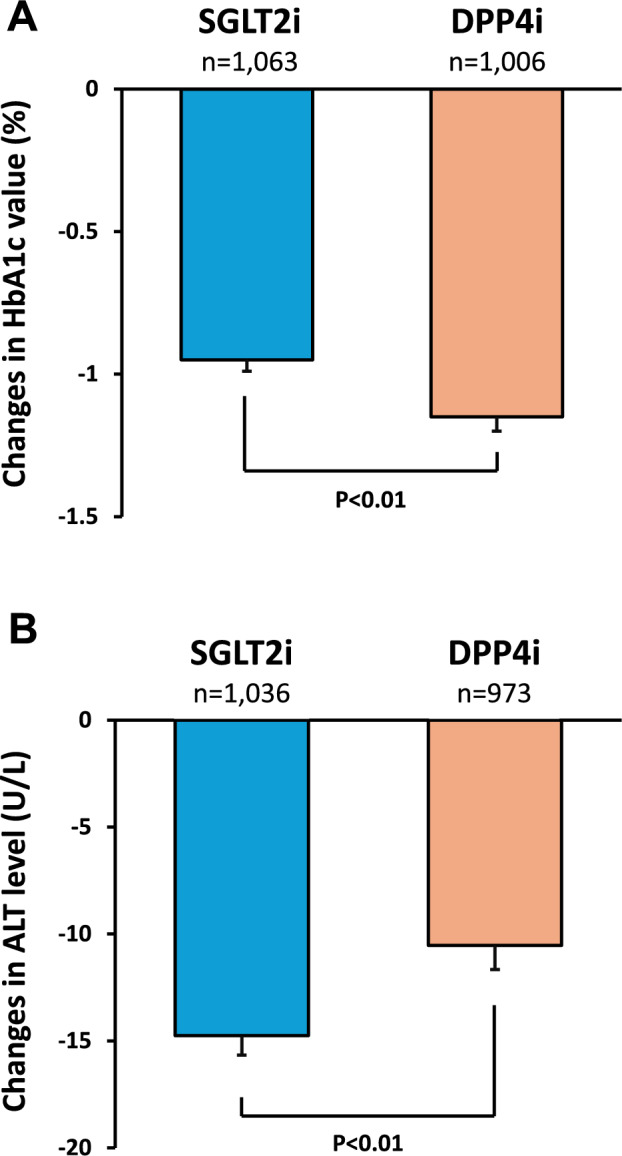
Effects of SGLT2i on HbA1c and ALT levels compared to DPP4i. **A** Changes in HbA1c level, **B** Changes in ALT level. Error bars represent standard error (SE). Significance was tested using Welch's t-test

Six months after treatment, SGLT2i and DPP4i decreased the HbA1c levels by 0.95% and 1.15%, respectively. DPP4i significantly decreased HbA1c levels compared to SGLT2i (Fig. 2A). In contrast, SGLT2i significantly decreased ALT levels compared to DPP4i 6 months after treatment (Fig. 2B). SGLT2i and DPP4i decreased ALT levels by 14.8 U/L and 10.5 U/L, respectively.

### Effects of SGLT2i in hepatic fibrosis indexes and DM-HCC risk score compared to DPP4i

**Fig. 3:**
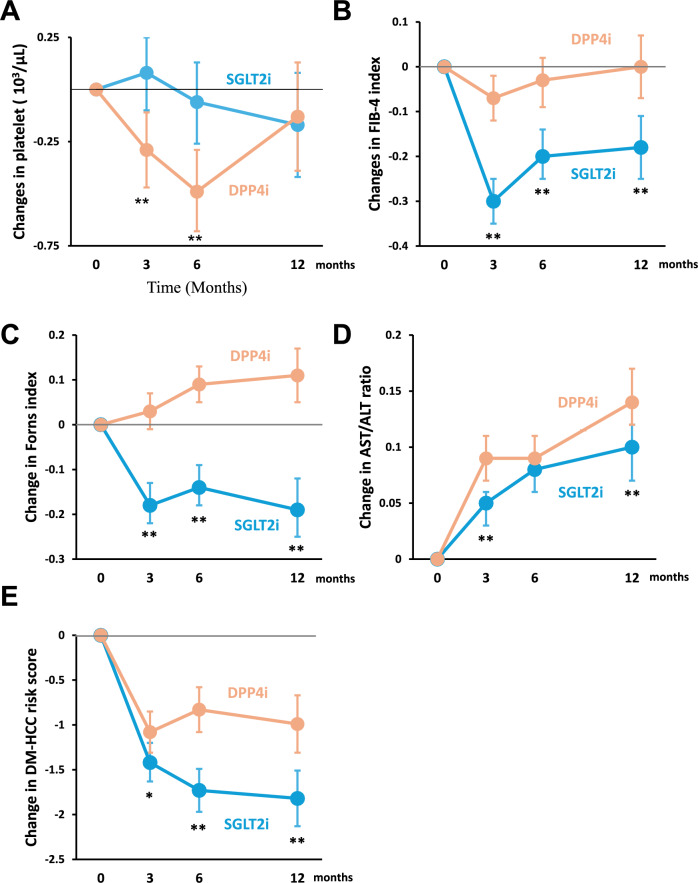
Effects of SGLT2i in hepatic fibrosis indexes and DM-HCC risk score compared to DPP4i. **A** Changes in platelet count, **B** Changes in FIB-4 index, **C** Changes in Forns index, **D** Changes in AST/ALT ratio, and **E** Changes in DM-HCC risk score. *P < 0.05, **P < 0.01. Intergroup comparison was conducted using the R package (lmerTest)

There was no significant difference in platelet counts between the two groups over 12 months (Fig. 3A). However, SGLT2i significantly improved the FIB-4 index, Forns index, and AST/ALT ratio than DPP4i over 12 months (Fig. 3B–D). In addition, SGLT2i significantly reduced the DM-HCC risk score compared to DPP4i over 12 months (Fig. 3E).

Supplementary Table 5 provides detailed statistical information on intragroup and intergroup differences in each hepatic fibrosis index and DM-HCC risk score at each time point during the 12 months.

### Effects of SGLT2i on the incidence of liver-related events compared to DPP4i

**Fig. 4:**
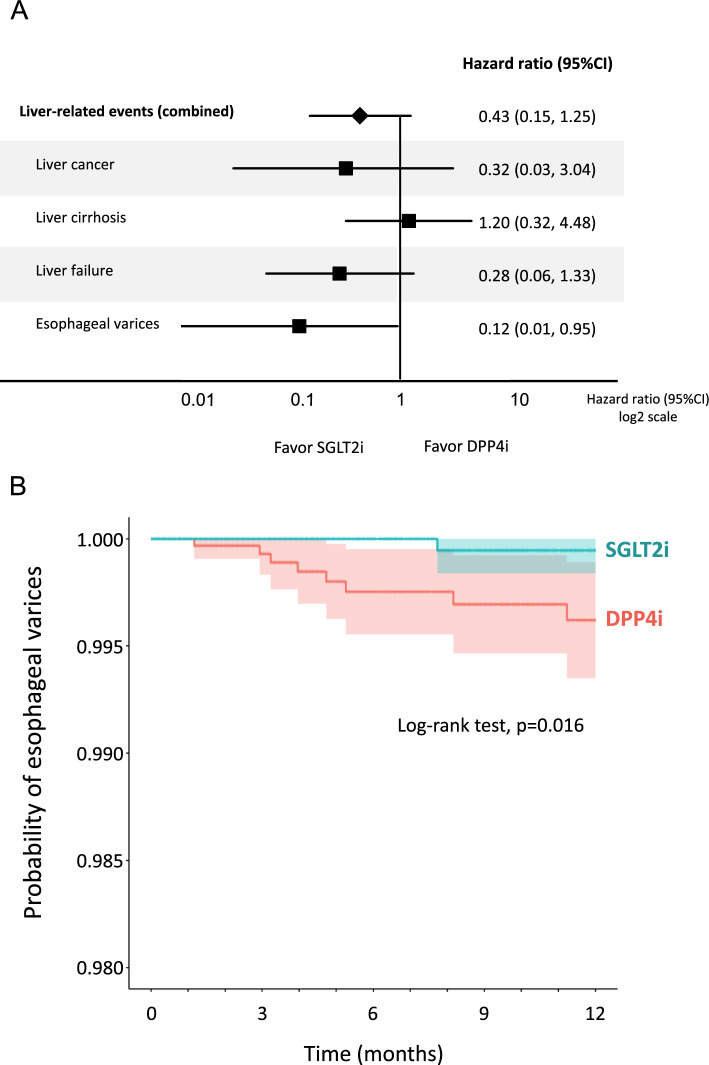
**A** Effects of SGLT2i on the incidence of liver-related events compared to DPP4i, **B** Kaplan–Meier curve for the incidence of esophageal varices in the SGLT2i group compared to the DPP4i group. Light-colored areas in each group indicate 95% CI

There was no significant difference in the overall incidence of liver-related events between the SGLT2i and DPP4i groups (Fig. 4A). In the analysis of each liver-related event, there was no significant difference in the incidence of liver cancer, liver cirrhosis, and liver failure between the two groups. However, SGLT2i significantly reduced the incidence of esophageal varices compared with DPP4i (Fig. 4A). Kaplan–Meier curve also demonstrated a significant reduction in the probability of esophageal varices in the SGLT2i group compared to the DPP4i group (Fig. 4B).

### Effects of SGLT2i on cardiovascular events compared to DPP4i

**Fig. 5:**
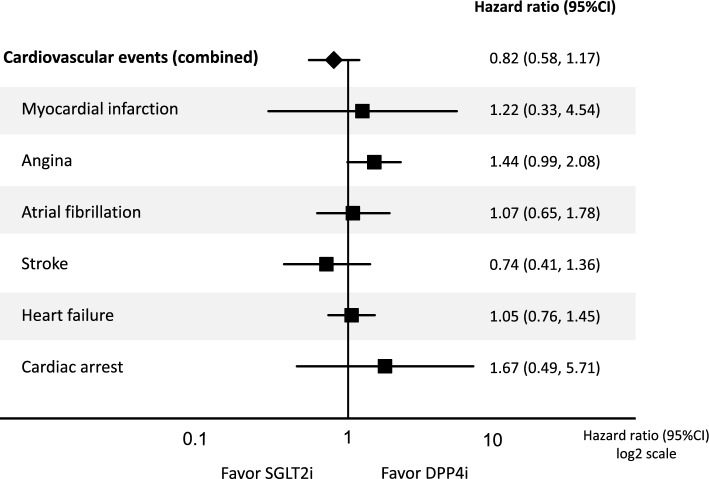
Effects of SGLT2i on cardiovascular events compared to DPP4i

There were no significant differences in cardiovascular events between the SGLT2i and DPP4i groups (Fig. 5). In the analysis of each cardiovascular event, there was no significant difference between the two groups in myocardial infarction, angina, atrial fibrillation, stroke, heart failure, and cardiac arrest (Fig. 5).

## Effects of SGLT2i on the incidence of extrahepatic cancer compared to DPP4i

**Fig. 6:**
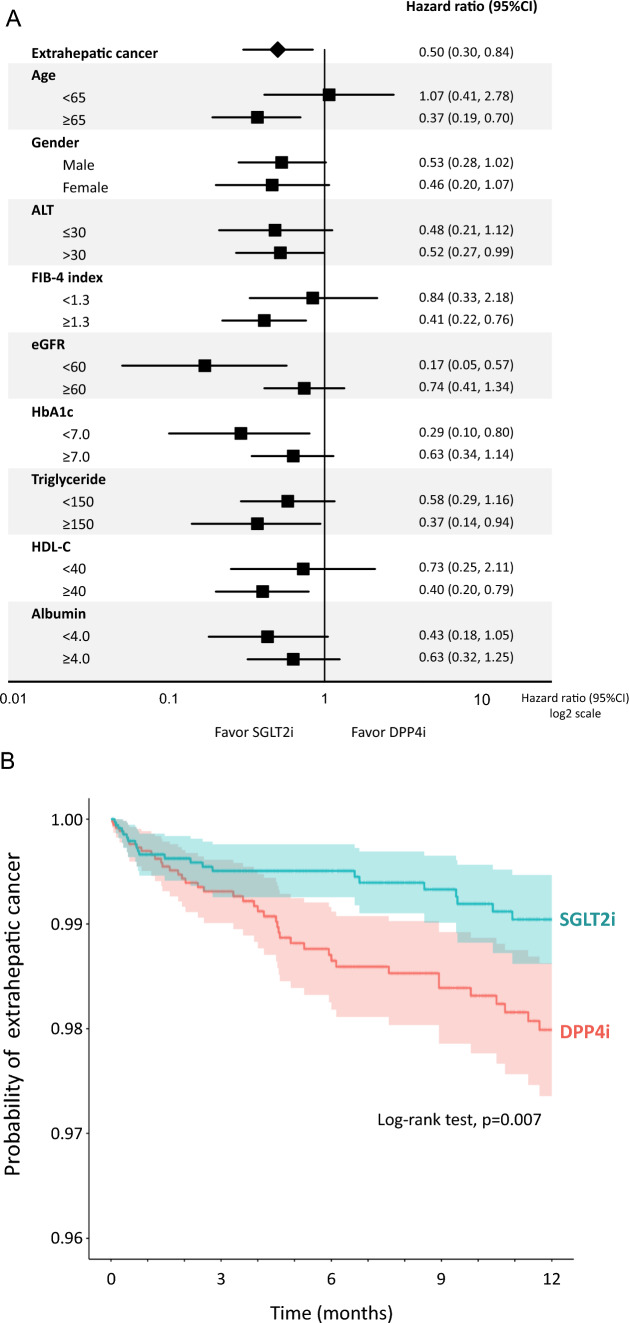
**A** Effects of SGLT2i on the incidence of extrahepatic cancer compared to DPP4i. **B** Kaplan–Meier curve for the incidence of extrahepatic cancer in the SGLT2i group compared to the DPP4i group. Light-colored areas in each group indicate 95% CI

SGLT2i significantly reduced the incidence of extrahepatic cancer compared to DPP4i (Fig. 6A). Kaplan–Meier curve also demonstrated a significant reduction in the probability of extrahepatic cancer in the SGLT2i group compared to the DPP4i group (Fig. 6B). We further performed a stratified analysis to identify patient characteristics associated with SGLT2i-caused suppression of the incidence of extrahepatic cancer. SGLT2i significantly suppressed the incidence of extrahepatic cancer in patients aged ≥ 65 years, ALT > 30 U/L, FIB-4 index ≥ 1.3, eGFR < 60 mL/min/1.73 m^2^, HbA1c < 7.0%, triglyceride ≥ 150 mg/dL, and HDL-C ≥ 40 mg/dL (Fig. 6A).

## Discussion

In this study, we demonstrated that SGLT2i was more effective than DPP4i in improving serum ALT levels and hepatic fibrosis indices in patients with T2DM and suspected MASLD. We also demonstrated that SGLT2i significantly decreased the incidence of esophageal varices compared with DPP4i. Moreover, SGLT2i significantly reduced the incidence of extrahepatic cancer compared to DPP4i in patients with T2DM and suspected MASLD.

Previous meta-analyses demonstrated that SGLT2i significantly decreased serum ALT levels in patients with T2DM and NAFLD [28, 29]. We further demonstrated that serum ALT levels were lower in the SGLT2i group than in the DPP4i group in this study. In contrast, HbA1c levels decreased to a greater extent in the DPP4i group than in the SGLT2i group. These findings suggest that the decrease in serum ALT levels by SGLT2i may be independent of glycemic control. Sumida et al. reported that SGLT2i reduced serum ALT levels and the reduction was correlated with the reduction of hepatic fat content [30]. In addition, SGLT2 is reported to occur in hepatocytes, and its expression is upregulated in hepatic steatosis [31, 32]. Moreover, in animal models of NAFLD, SGLT2i inhibits hepatic SGLT2 and reduces serum ALT levels through an upregulation of autophagy via the AMP-activated protein kinase-mammalian target of rapamycin pathway in hepatocytes [33, 34]. Thus, a decrease in serum ALT levels by SGLT2i may be partly caused by an improvement in hepatic steatosis through the induction of autophagy in hepatocytes.

We found that SGLT2i was more effective than DPP4i in improving various hepatic fibrosis indices, including the FIB-4 and Forns indices. Takahashi et al. investigated the effects of SGLT2i on liver histology of patients with T2DM and MASLD in a randomized controlled trial [35]. They performed repeated liver biopsies and demonstrated that SGLT2i significantly improved hepatic fibrosis compared to other diabetic medications besides SGLT2i. In addition, meta-analyses have demonstrated that in patients with T2DM and MASLD, SGLT2i significantly improves non-invasive hepatic fibrosis indices, including liver stiffness measurement [8, 36, 37]. Moreover, Shen et al. demonstrated that SGLT2i ameliorates MASLD-associated fibrosis by downregulating miR-34a-5p and targeting *GREM2* to inhibit the transforming growth factor β pathway in hepatic stellate cells [38]. Thus, our findings are in good agreement with previous clinical and basic studies. SGLT2i may be more beneficial in improving hepatic fibrosis than DPP4i in patients with T2DM and suspected MASLD.

We first demonstrated that the incidence of esophageal varices was significantly lower in the SGLT2i group than in the DPP4i group. In contrast, Hsu et al. reported that empagliflozin, an SGLT2i, did not ameliorate portal hypertension in cirrhotic rats [39]. This discrepancy may be due to the different etiologies of liver disease. A previous study used a biliary cirrhosis model, but not a MASLD model. In our study, SGLT2i significantly improved the risk factors for esophageal varices, such as serum ALT levels and hepatic fibrosis indices. Besides, SGLT2 inhibitors reduce fluid volume, which may contribute to the reduction of portal vein pressure. Moreover, Simon et al. demonstrated that the incidence of esophageal variceal hemorrhage was significantly lower in the SGLT2i group or GLP-1 receptor agonist group compared to that in the DPP4i and sulfonylurea groups in patients with T2DM and liver cirrhosis [40]. This previous study could not directly compare the incidence of esophageal variceal hemorrhage between the SGLT2i and DPP4i groups due to the small sample size. However, these findings suggest that SGLT2i might be superior to DPP4i in lowering the incidence of esophageal variceal hemorrhage. Taken together, SGLT2i seems to be more beneficial than DPP4i in inhibiting the onset of esophageal varices in patients with T2DM and suspected MASLD.

In this study, the SGLT2i group showed a significantly lower incidence of extrahepatic cancer than the DPP4i group. The beneficial effects of SGLT2i were evident in patients at high risk of cancer, including the elderly, hepatitis, hepatic fibrosis, chronic kidney disease, and hypertriglyceridemia. We have to be cautious about the interpretation of the results. Because the MDV database does not include the details of medical history, there is a possible bias in the duration of T2DM history between the DPP4i and SGLT2i groups. In addition, we could not examine specific organs affected by cancer. However, in clinical studies, SGLT2i has been reported to suppress the incidence of colorectal and breast cancers compared to DPP4i [16, 41]. Moreover, Tanaka et al. demonstrated that SGLT2i significantly suppressed the incidence of pancreatic cancer using a 15-year Japan Medical Data Center administrative claims database [42]. These cancer types are highly prevalent in patients with T2DM, supporting our results. In basic studies, expression of SGLT2 has been observed in human colorectal, breast, and pancreatic cancer cells [13, 43, 44]. Moreover, SGLT2i has been reported to block glucose uptake into cancer cells and suppress tumor growth[13, 14]. Furthermore, SLGT2i has several mechanisms to suppress cancer cells, including mitochondrial membrane instability, suppression of β-catenin, and downregulation of ATP production [15, 45]. These previous studies provide theoretical mechanisms by which SGLT2i suppresses carcinogenesis and support our results.

We demonstrated a significant reduction in the incidence of extrahepatic cancer in the SGLT2i group compared to the DPP4i group over 12 months. For assessing the chemo-preventive effects of drugs on carcinogenesis, the observation period is generally greater than 3 years. Thus, the issue of the observational period exists in the evaluation of the incidence of extrahepatic cancer. Conversely, Chung et al. recently reported that SGLT2i significantly suppressed the onset of HCC than DPP4i using the Korean National Health Insurance Service database [46]. The suppression was observed from 6 months after SGLT2i treatment and becomes obvious at one year. In addition, there have been randomized controlled phase 3 clinical trials evaluating the impact of SGLT2i on adverse events including carcinogenesis over 1 year [47, 48]. These previous studies demonstrated a lower incidence of malignancies in the SGLT2i group than in the placebo group. These previous studies may support our findings that SGLT2i suppressed the incidence of extrahepatic malignancies than DPP4i even at 1 year. Cancer is becoming the leading cause of death in patients with T2DM [49]. Therefore, SGLT2i is expected to contribute to improving survival by suppressing the incidence of cancer in patients with T2DM.

This study had several limitations. First, this was an observational study, and we cannot deny the possibility of the presence of unevaluated confounding factors. Second, the MDV database did not include data on body mass index, abdominal circumference, and imaging information. Therefore, we defined suspected MASLD as a predefined cutoff for ALT values for Japanese patients with NAFLD[21] and excluded other liver diseases. We also evaluated hepatic fibrosis using noninvasive indices, but not liver biopsy. Third, the observational period was 12 months and the incidence of liver-related events, cardiovascular events, and extrahepatic cancer must be verified with a long-term follow-up study. Thus, the effects of SGLT2i on prognosis should be evaluated in a prospective randomized controlled trial using imaging modalities with a long-term follow-up period.

In conclusion, SGLT2i was more effective than DPP4i in improving serum ALT levels and hepatic fibrosis indices in patients with T2DM and suspected MASLD. In addition, SGLT2i significantly decreased the incidence of esophageal varices compared with DPP4i. Moreover, SGLT2i significantly reduced the incidence of extrahepatic cancer compared with DPP4i. These findings suggest that SGLT2i may be more beneficial than DPP4i in suppressing life-threatening events in patients with T2DM and suspected MASLD.

## Supplementary Information

Below is the link to the electronic supplementary material.

Supplementary file1 (DOCX 40 KB)
