# Supplementary material for: Effects of SGLT2 inhibitors on the onset of esophageal varices and extrahepatic cancer in type 2 diabetic patients with suspected MASLD: a nationwide database study in Japan
Source: J Gastroenterol. 2024 Oct 11;59(12):1120–32. doi: 10.1007/s00535-024-02158-z (PMC11541318; doi:10.1007/s00535-024-02158-z)
Supplement: Supplementary file 1 — Supplementary file1 (DOCX 40 KB) [file 535_2024_2158_MOESM1_ESM.docx]

Supplementary Table 1. ICD-10 codes used for exclusion criteria, pre-existing conditions, liver-related events, and cardiovascular events

| **Definition** | **ICD-10 codes** |
| --- | --- |
| **Excluding criteria: Diabetes mellitus** |  |
| Type 1 diabetes mellitus | E10 |
| Malnutrition-related diabetes mellitus | E12 |
| Other specified diabetes mellitus | E13 |
| Diabetes mellitus in pregnancy | O24 |
| **Excluding criteria: Disease associated with abnormal liver function tests other than MASLD/MASH** | |
| Alcohol-related liver disease | K70 |
| Viral hepatitis | B16-B19 |
| Primary biliary cirrhosis | K74.3 |
| Autoimmune hepatitis | K75.4 |
| Primary sclerosing cholangitis | K83.0 |
| Hemochromatosis | E83.1 |
| Wilson's disease | E83.0 |
| Alpha-1-antitrypsin deficiency | E88.0 |
| Budd-Chiari syndrome | I82.0 |
| Secondary or unspecified biliary cirrhosis | K74.4, K74.5 |
| Codes associated with alcohol use disorder | F10 |
| Codes associated with somatic consequences of alcohol (except alcohol-related liver disease) | E24.4, G31.2, G62.1, G72.1, I42.6, K29.2, K85.2, K86.0, T51.0, T51.9 |
| Codes associated with drug use disorders except nicotine/caffeine | F11, F12, F13, F14, F16, F18, F19 |
| Acquired immune deficiency syndrome/ Human immunodeficiency virus | B20, B21, B22, B23, B24 |
| Toxic liver disease (including drug-induced liver disease) | K71 |
| Cholecystitis/Cholangitis | K81, K83 |
| Liver cancer (including secondary liver cancer) | C22, C78.7 |
| **Pre-existing condition** |  |
| Hypertension | I10-I15 |
| Lipoprotein metabolism disorder | E78 |
| Chronic hepatitis | K73.2, K73.9 |
| Liver cirrhosis/Ascites | K74.6, K76.6, K76.7, R18 |
| **Liver-related events** |  |
| Hepatic failure | K72 |
| Esophageal varices | I85, I86.4 |
| Liver cirrhosis/Ascites | K74.6, K76.7, R18 |
| Liver cancer | C22 |
| Extrahepatic cancer | C00-C21, C23-C96 |
| **Cardiovascular events** |  |
| Heart failure | I50 |
| Cardiac arrest | I46 |
| Stroke | I60-I64 |
| Myocardial infarction | I21 |
| Angina pectoris | I20 |
| Atrial fibrillation | I48 |

Supplementary Table 2. Covariates for propensity score matching

| **Covariate** | | **Type** |
| --- | --- | --- |
| Sex | | Binary |
| Age | | Count |
| Year of Index date | 2014 | Binary |
|  | 2015 |  |
|  | 2016 |  |
|  | 2017 |  |
|  | 2018 |  |
|  | 2019 |  |
|  | 2020 |  |
|  | 2021 |  |
|  | 2022 |  |
| Hospitalized | | Binary |
| Total hospital days | | Count |
| With/without diabetes medication prescription (by drug class) at the index date | α-glucosidase inhibitor | Binary |
|  | Sulfonylureas |  |
|  | GLP-1 receptor agonists |  |
|  | Glitazones |  |
|  | Insulin |  |
|  | Glinides |  |
|  | Biguanides |  |
|  | Other antidiabetic |  |
| Number of diabetes medications at index date | | Count |
| Laboratory tests value (baseline) | HbA1c | Continuous variable |
|  | ALT |  |
|  | AST |  |
|  | Platelet |  |
|  | γ-GTP |  |
|  | Triglyceride |  |
|  | Total cholesterol |  |
|  | eGFR |  |
| Hepatic fibrosis index (baseline) | FIB-4 index | Continuous variable |
|  | Forns index |  |
|  | APRI |  |
|  | Age-Platelet index |  |
|  | AST/ALT ratio |  |
|  | DM-HCC risk score |  |
| With/without concomitant medications | Diuretics | Binary |
|  | Beta-blockers |  |
|  | Calcium antagonists |  |
|  | Angiotensin-converting enzyme inhibitor/angiotensin receptor blocker |  |
|  | Other antihypertensives |  |
|  | Statin |  |
|  | Fibrates |  |
|  | Vitamin E |  |
|  | Other anti-hyperlipidemics |  |
| With/without concomitant disease as defined by Charlson Comorbidity Index (CCI) | Chronic hepatitis | Binary |
|  | Hepatic cirrhosis |  |
|  | Dyslipidemia |  |
|  | Hypertension |  |
|  | Acquired immune deficiency syndrome/ Human immunodeficiency virus |  |
|  | Any malignancy, including lymphoma and leukemia, except malignant neoplasm of skin |  |
|  | Cerebrovascular disease |  |
|  | Chronic obstructive pulmonary disease |  |
|  | Congestive heart failure |  |
|  | Dementia |  |
|  | Diabetes with complications |  |
|  | Diabetes without complications |  |
|  | Hemiplegia or paraplegia |  |
|  | Metastatic solid tumor |  |
|  | Mild liver disease |  |
|  | Moderate or severe liver disease |  |
|  | Myocardial infarction |  |
|  | Peptic ulcer disease |  |
|  | Peripheral vascular disorders |  |
|  | Renal disease |  |
|  | Rheumatic disease |  |
| Probabilities of censoring (inverse probability of censoring weighting) | | Continuous variable |

Supplementary Table 3. ATC codes for concomitant medications

| **Definition** | **ATC codes** |
| --- | --- |
| **Concomitant diabetes medications** |  |
| Insulin | A10C |
| Sulfonylureas | A10H |
| Biguanides | A10J |
| Glitazones | A10K |
| α-glucosidase inhibitor | A10L |
| Glinides | A10M |
| GLP-1 receptor agonists | A10S |
| Other anti-diabetics | A10X |
| **Other concomitant medications** |  |
| Anti-hypertensives | C02 |
| Diuretics | C03 |
| Beta-blockers | C07 |
| Calcium antagonists | C08 |
| Angiotensin-converting enzyme inhibitor/angiotensin receptor blocker | C09 |
| Statins | C10A1 |
| Vitamin E | A11X3 |
| Fibrates | C10A2 |
| Other anti-hyperlipidemics | C10A3, C10A4, C10A9, C10B, C10C |

Supplementary Table 4

| **Item** | **Formula** |
| --- | --- |
| FIB-4 index | (AST (U/L) × Age (years))/(Platelet (10^9^/L) ×ALT (U/L)^1/2^) |
| Forns index | 7.811-3.131 log Platelet (10^9^/L)+0.781 log γ-GTP (U/L)+3.467 log Age (years)-0.014 Total cholesterol (mg/dL) |
| APRI | 100× (AST (U/L)/30)/PLT (10^9^/L) |
| Age-Platelet index | Age score+Platelet score *Age score* <30: 0, 30-39: 1, 40-49: 2, 50-59: 3, 60-69: 4, ≥70: 5 *Platelet score* ≥ 225: 0; 200-224: 1; 175-199: 2; 150-174: 3; 125-149: 4; < 125: 5 |
| AST/ALT ratio | AST (U/L)/ALT (U/L) |
| DM-HCC risk score | Age score+γ-GTP score+Triglyceride score *Age score* >65: +11, ≤65: 0 *γ-GTP score* >80 (U/L): +16, 41-80 (U/L): +8, ≤40: 0  *Triglyceride score* <150(mg/dL): +6, ≥150 (mg/dL): 0 |

Supplementary Table 5. Intragroup and intergroup differences in each hepatic fibrosis index and DM-HCC risk score at each time point during the 12 months

|  |  |  | SGLT2i | |  | DPP4i | |  | Estimate of difference (SGLT2i - DPP4i) (95%CI) | p-value** |
| --- | --- | --- | --- | --- | --- | --- | --- | --- | --- | --- |
|  | Months |  | Estimate of change (95%CI) | p-value* |  | Estimate of change (95%CI) | p-value* |  |  |  |
| HbA1c (%) | 3 |  | -0.94 (-0.99, -0.88) | <0.001 |  | -1.23 (-1.29, -1.17) | <0.001 |  | 0.29 (0.21, 0.37) | <0.001 |
|  | 6 |  | -0.95 (-1.01, -0.89) | <0.001 |  | -1.16 (-1.22, -1.1) | <0.001 |  | 0.21 (0.12, 0.3) | <0.001 |
|  | 12 |  | -1.02 (-1.09, -0.94) | <0.001 |  | -1.02 (-1.1, -0.94) | <0.001 |  | 0 (-0.11, 0.11) | 0.994 |
| ALT (U/L) | 3 |  | -14.5 (-17.16, -11.84) | <0.001 |  | -13.05 (-15.8, -10.3) | <0.001 |  | -1.45 (-5.27, 2.38) | 0.458 |
|  | 6 |  | -14.67 (-17.62, -11.71) | <0.001 |  | -10.44 (-13.47, -7.41) | <0.001 |  | -4.23 (-8.46, 0.01) | 0.05 |
|  | 12 |  | -16.16 (-19.98, -12.34) | <0.001 |  | -10.93 (-14.86, -7) | <0.001 |  | -5.23 (-10.71, 0.26) | 0.062 |
| AST (U/L) | 3 |  | -12.26 (-14.53, -9.98) | <0.001 |  | -7.86 (-10.22, -5.5) | <0.001 |  | -4.4 (-7.68, -1.11) | 0.009 |
|  | 6 |  | -10.27 (-12.81, -7.74) | <0.001 |  | -6.34 (-8.95, -3.73) | <0.001 |  | -3.94 (-7.58, -0.3) | 0.034 |
|  | 12 |  | -11.15 (-14.43, -7.86) | <0.001 |  | -6.77 (-10.16, -3.38) | <0.001 |  | -4.38 (-9.1, 0.34) | 0.069 |
| γGTP (U/L) | 3 |  | -19.17 (-21.41, -16.93) | <0.001 |  | -18.07 (-20.4, -15.73) | <0.001 |  | -1.11 (-4.34, 2.13) | 0.502 |
|  | 6 |  | -17.89 (-20.38, -15.39) | <0.001 |  | -12.19 (-14.76, -9.62) | <0.001 |  | -5.7 (-9.28, -2.11) | 0.002 |
|  | 12 |  | -18.65 (-21.88, -15.42) | <0.001 |  | -13.09 (-16.44, -9.75) | <0.001 |  | -5.56 (-10.21, -0.91) | 0.019 |
| eGFR | 3 |  | -2.54 (-2.95, -2.13) | <0.001 |  | -3.21 (-3.64, -2.79) | <0.001 |  | 0.68 (0.09, 1.27) | 0.024 |
|  | 6 |  | -2.41 (-2.86, -1.95) | <0.001 |  | -3.06 (-3.53, -2.6) | <0.001 |  | 0.65 (0, 1.31) | 0.049 |
|  | 12 |  | -2.24 (-2.83, -1.65) | <0.001 |  | -4.5 (-5.1, -3.89) | <0.001 |  | 2.26 (1.41, 3.1) | <0.001 |
| Triglyceride (mg/dL) | 3 |  | -17.89 (-23.57, -12.21) | <0.001 |  | -29.68 (-35.69, -23.67) | <0.001 |  | 11.79 (3.52, 20.06) | 0.005 |
|  | 6 |  | -16.93 (-23.19, -10.67) | <0.001 |  | -20.24 (-26.8, -13.69) | <0.001 |  | 3.31 (-5.75, 12.38) | 0.474 |
|  | 12 |  | -21.85 (-29.91, -13.79) | <0.001 |  | -11.75 (-20.21, -3.3) | 0.006 |  | -10.1 (-21.78, 1.59) | 0.09 |
| Total cholesterol (mg/dL) | 3 |  | -6.04 (-7.89, -4.2) | <0.001 |  | -10.75 (-12.51, -8.98) | <0.001 |  | 4.7 (2.15, 7.25) | <0.001 |
|  | 6 |  | -4.26 (-6.31, -2.21) | <0.001 |  | -11.01 (-12.98, -9.04) | <0.001 |  | 6.76 (3.91, 9.6) | <0.001 |
|  | 12 |  | -2.83 (-5.54, -0.11) | 0.041 |  | -12.46 (-15.01, -9.91) | <0.001 |  | 9.63 (5.91, 13.36) | <0.001 |
| Platelet (×10,000/μL) | 3 |  | 0.08 (-0.1, 0.25) | 0.385 |  | -0.29 (-0.47, -0.11) | 0.002 |  | 0.36 (0.11, 0.61) | 0.005 |
|  | 6 |  | -0.06 (-0.26, 0.13) | 0.54 |  | -0.49 (-0.68, -0.29) | <0.001 |  | 0.42 (0.15, 0.7) | 0.003 |
|  | 12 |  | -0.17 (-0.42, 0.08) | 0.185 |  | -0.13 (-0.39, 0.13) | 0.313 |  | -0.04 (-0.4, 0.33) | 0.843 |
| FIB-4 index | 3 |  | -0.3 (-0.35, -0.25) | <0.001 |  | -0.07 (-0.12, -0.02) | 0.006 |  | -0.23 (-0.3, -0.16) | <0.001 |
|  | 6 |  | -0.2 (-0.25, -0.14) | <0.001 |  | -0.03 (-0.09, 0.02) | 0.254 |  | -0.16 (-0.24, -0.09) | <0.001 |
|  | 12 |  | -0.18 (-0.25, -0.11) | <0.001 |  | 0 (-0.07, 0.07) | 0.997 |  | -0.18 (-0.28, -0.08) | <0.001 |
| Forns index | 3 |  | -0.18 (-0.22, -0.13) | <0.001 |  | 0.03 (-0.01, 0.07) | 0.156 |  | -0.2 (-0.26, -0.15) | <0.001 |
|  | 6 |  | -0.14 (-0.18, -0.09) | <0.001 |  | 0.09 (0.05, 0.13) | <0.001 |  | -0.23 (-0.29, -0.17) | <0.001 |
|  | 12 |  | -0.19 (-0.25, -0.12) | <0.001 |  | 0.11 (0.05, 0.17) | <0.001 |  | -0.3 (-0.38, -0.21) | <0.001 |
| AST/ALT | 3 |  | 0.05 (0.03, 0.06) | <0.001 |  | 0.09 (0.07, 0.11) | <0.001 |  | -0.04 (-0.07, -0.02) | <0.001 |
|  | 6 |  | 0.08 (0.06, 0.09) | <0.001 |  | 0.09 (0.08, 0.11) | <0.001 |  | -0.02 (-0.04, 0.01) | 0.181 |
|  | 12 |  | 0.1 (0.07, 0.12) | <0.001 |  | 0.14 (0.12, 0.17) | <0.001 |  | -0.05 (-0.08, -0.01) | 0.006 |
| DM-HCC risk score | 3 |  | -1.42 (-1.63, -1.2) | <0.001 |  | -1.08 (-1.31, -0.85) | <0.001 |  | -0.34(-0.65, -0.02) | 0.036 |
|  | 6 |  | -1.73 (-1.97, -1.49) | <0.001 |  | -0.83 (-1.08, -0.58) | <0.001 |  | -0.89 (-1.24, -0.55) | <0.001 |
|  | 12 |  | -1.82 (-2.13, -1.51) | <0.001 |  | -0.99 (-1.31, -0.67) | <0.001 |  | -0.83 (-1.27, -0.38) | <0.001 |

*: Intragroup comparison from baseline was conducted using R package (lmerTest)

**: Intergroup comparison was conducted using R package (lmerTest)
